# Clinician Distribution and Type in Rural and Urban Areas of the National Health Services Corps

**DOI:** 10.1001/jamanetworkopen.2024.45995

**Published:** 2024-11-19

**Authors:** Olesya Baker, Marcela Horvitz-Lennon, Hao Yu

**Affiliations:** 1 Department of Population Medicine, Harvard Medical School and Harvard Pilgrim Health Care Institute, Boston, Massachusetts; 2 RAND Corporation, Boston, Massachusetts

## Abstract

This cross-sectional study assesses long-term trends in clinician distribution between rural and urban areas and whether the trends differ by clinician discipline and type.

## Introduction

The US faces severe shortages of health professionals, with rural areas disproportionately affected.^1,2^ Their impact on rural residents’ health care access and contribution to rural-urban disparities in health outcomes^3,4^ underscore the significance of policies designed to expand the rural clinical workforce.

This is not a new problem. Over 50 years ago, Congress created the National Health Services Corp (NHSC) to address clinician shortages. NHSC provides financial incentives to clinicians who commit to practice in health professional shortage areas (HPSAs) designated by the Health Resources and Services Administration (HRSA). HPSA designations identify shortage areas across 3 disciplines: primary care, dental care, and mental health care. In addition to recruiting new clinicians, in recent years the NHSC has prioritized retaining health professionals who already practice in HPSAs. While the NHSC workforce has substantially expanded since 2009, the immediate changes in the proportion of NHSC clinicians practicing in rural areas were modest.^5^ This study expands the literature by assessing long-term trends in the NHSC clinician distribution between rural and urban areas and whether the trends differ by clinician discipline and type.

## Methods

This cross-sectional study was deemed nonhuman participants research by the institutional review board at Harvard Medical School and informed consent was not required. This study followed the Strengthening the Reporting of Observational Studies in Epidemiology (STROBE) reporting guideline. This repeated cross-sectional study used the 2000 to 2020 HRSA NHSC Field Strength Database containing information on clinician’s practice location (rural vs urban), discipline (primary care, mental health care, and dental care) and type (physicians vs nonphysicians) (eTable in Supplement 1). To account for changes in HPSA population size, we used the HRSA 2000 to 2020 HPSA Quarterly Summary. We examined the annual percentage of NHSC clinicians in rural areas by clinician discipline and type and analyzed changes in NHCS clinician density (ie, number of NHSC clinician per 100 000 HPSA population) by discipline and rurality. All analyses were conducted using Stata SE version 16.0 (StataCorp). Data were analyzed from February 2024 to September 2024.

## Results

**Figure 1.**
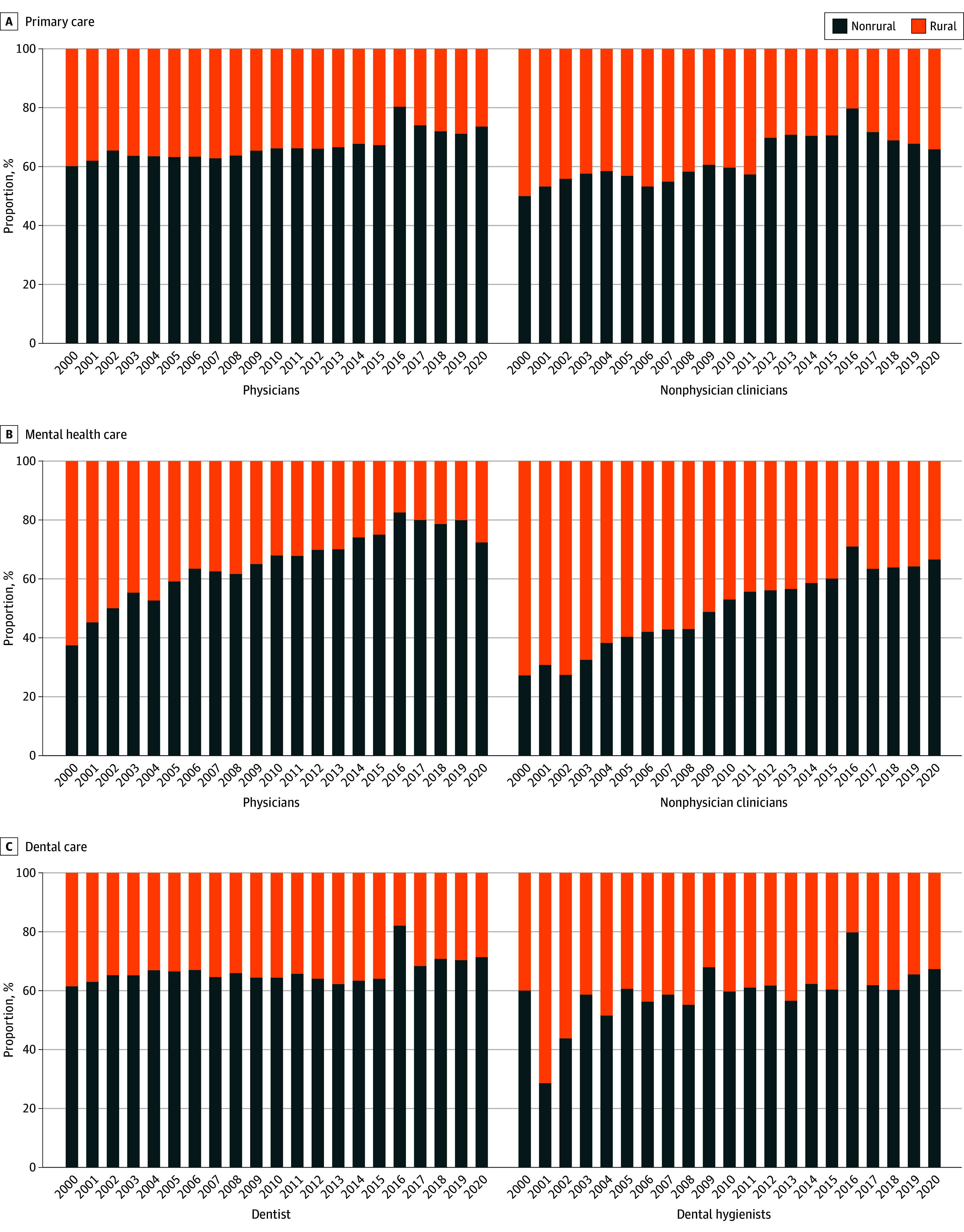
Percentages of the National Health Services Corp (NHSC) Health Care Clinicians Practicing in Rural Areas, 2000-2020 Percentages were calculated by aggregating clinician full-time equivalent from the NHSC Field Strength Database and computing urban or rural percentages for each year and clinician type. The rural status variable is defined by the Health Resources and Services Administration and includes: all nonmetro counties, all metro census tracts with rural-urban commuting area codes (RUCA) codes 4 to 10 and large area metropolitan census tracts of at least 400 square miles in area with population density of 35 or less per square mile with RUCA codes 2 and 3.

**Figure 2.**
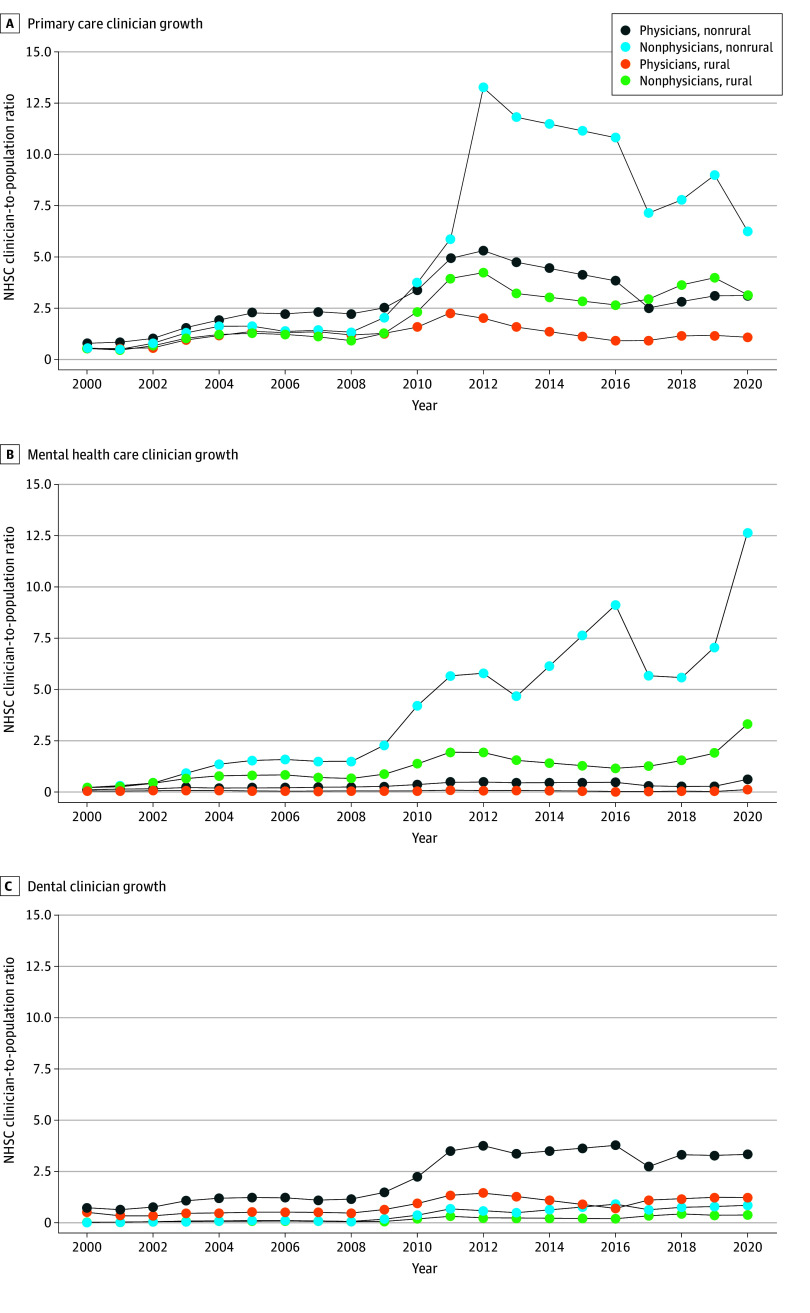
Growth of the National Health Services Corp (NHSC) Health Care Clinicians per 100 000 Health Professional Shortage Area (HPSA) Population by Clinician Type and Geography NHSC clinician-to-population ratio was computed as total number of NHSC clinicians in a given year divided by total population of designated HPSAs in that year, stratified by HRSA rural-urban status. We did not include 2014 and 2015 because the HRSA’s HPSA Quarterly Summary was unavailable for these 2 years, leading to missing information about HPSA populations. The Quarterly Summaries categorized rural vs urban areas as metropolitan and nonmetropolitan before 2014, and as rural, partially rural, and nonrural after 2016. We standardized the rurality definition across years by classifying an HPSA as rural if it was listed as nonmetropolitan, partially rural, or rural and as urban if metropolitan or nonrural.

The number of NHSC clinicians grew from 900 to 15 637 between 2000 and 2020. While the difference in the number of NHSC clinicians practicing in rural vs urban areas was small in 2000 (428 [48%] vs 472 [52%]), it was large in 2020 (5049 [32%] vs 10 588 [68%]). The proportion of NHSC clinicians in rural areas declined between 2000 and 2020 across all 3 disciplines for both physicians and nonphysicians, with rural mental health care nonphysicians experiencing the largest decline (Figure 1). From 2000 until the 2009 NHSC expansion, the NHSC clinician density was relatively flat and the rural-urban gap widened only slightly. Across all specialties, rural-urban gap in clinician density was 0.15 in 2000, 0.9 in 2009, and 3.1 by 2020. The gap increased dramatically after the expansion, as the trend in NHSC clinician density across the 3 disciplines remained largely flat for rural physicians and increased slightly for rural nonphysicians while urban clinician density increased sharply, especially among urban primary and mental health care nonphysicians and dentists (Figure 2).

## Discussion

The 2009 NHSC expansion increased the overall number of NHSC clinicians in HPSAs but also widened the rural-urban imbalance in their distribution. The HRSA used HPSA scores to determine workforce needs, prioritizing areas with higher HPSA scores for NHSC clinician hiring. However, because scores were blind to rural status, rural sites’ geographic isolation, greater housing shortages, and limited health care infrastructure likely affect clinicians’ preference for rural positions.^1,6^ Policies to reduce the observed rural-urban disparities should prioritize NHSC recruitment and retention in rural areas.

Study limitations were the use of total HPSA population by rural-urban status, which does not capture complex within-area differences in health care needs, and a lack of longitudinal data on HPSA scores across all NHSC-approved sites, limiting our ability to assess health care need changes by rural-urban status. Future studies should assess the contribution of the NHSC clinician distribution to rural-urban disparities in health care and health outcomes.

## Notes

Supplement 1. **eTable.** Classification of Clinician Types into Physician and Nonphysician Categories

Supplement 2.
Data Sharing Statement
