# Supplementary material for: Clinician Distribution and Type in Rural and Urban Areas of the National Health Services Corps
Source: JAMA Netw Open. 2024 Nov 19;7(11):e2445995. doi: 10.1001/jamanetworkopen.2024.45995 (PMC11577137; doi:10.1001/jamanetworkopen.2024.45995)
Supplement: Supplement 1. — eTable. Classification of Clinician Types into Physician and Nonphysician Categories [file jamanetwopen-e2445995-s001.pdf]

Supplemental Online Content

Baker O, Horvitz-Lennon M, Yu H. Clinician distribution and type in rural and urban areas of the National Health Services Corps. *JAMA Netw Open*. 2024;7(11):e2445995. doi:10.1001/jamanetworkopen.2024.45995

eTable. Classification of Clinician Types into Physician and Nonphysician Categories

This supplemental material has been provided by the authors to give readers additional information about their work.

© 2024 Baker O et al. *JAMA Network Open*.

eTable. Classification of Clinician Types into Physician and Nonphysician Categories

Clinician Type	Specialty
**Primary Care Physicians**	
Allopathic Physician	Family Practice
Allopathic Physician	Family Practice - Geriatrics
Allopathic Physician	Family Practice w/OB
Allopathic Physician	Internal Medicine
Allopathic Physician	Internal Medicine - Geriatrics
Allopathic Physician	None
Allopathic Physician	OB/GYN
Allopathic Physician	Pediatrics
Osteopathic Physician	Family Practice
Osteopathic Physician	Family Practice - Geriatrics
Osteopathic Physician	Family Practice w/OB
Osteopathic Physician	General Practice
Osteopathic Physician	Internal Medicine
Osteopathic Physician	Internal Medicine - Geriatrics
Osteopathic Physician	OB/GYN
Osteopathic Physician	Pediatrics
**Primary Care Non-Physician Clinicians**	
Chiropractor	None
Clinical Nurse Specialist	None
Nurse Practitioner	Adult
Nurse Practitioner	Family Practice
Nurse Practitioner	Geriatrics
Nurse Practitioner	None
Nurse Practitioner	Pediatrics
Nurse Practitioner	Womens Health
Pharmacist	None
Physician Assistant	Adult
Physician Assistant	Family Practice
Physician Assistant	None
Physician Assistant	Pediatrics
Pharmacist	None
Physician Assistant	Adult
Physician Assistant	Family Practice
Physician Assistant	None
Physician Assistant	Pediatrics
Physician Assistant	Womens Health
Registered Nurse	None
Registered Nurse Anesthetist	None

© 2024 Baker O et al. *JAMA Network Open*.

Nurse Anesthetist	None
Certified Nurse Anesthetist	None
Certified Nurse Midwife	None
**Mental Health Care Physicians**	
Allopathic Physician	Mental Health
Osteopathic Physician	Mental Health
**Mental Health Care Non-Physician Clinicians**	
Health Service Psychologist	None
Licensed Clinical Social Worker	None
Licensed Professional Counselor	None
Marriage and Family Therapist	None
Nurse Practitioner	Psychiatry
Physician Assistant	Psychiatry
Psychiatric Nurse Specialist	Adult
Psychiatric Nurse Specialist	None
Psychiatric Nurse Specialist	Pediatrics
Substance Use Disorder Counselor	None
State Licensed or Certified SUD Counselor	None
**Dentists**	
Dentist	General Practice
Dentist	Geriatrics
Dentist	None
Dentist	Pediatrics
Dentist	Public Health Dentistry
**Dental Hygienist**	
Registered Dental Hygienist	None

© 2024 Baker O et al. *JAMA Network Open*.
