# Supplementary material for: Clinician Distribution and Type in Rural and Urban Areas of the National Health Services Corps
Source: JAMA Netw Open. 2024 Nov 19;7(11):e2445995. doi: 10.1001/jamanetworkopen.2024.45995 (PMC11577137; doi:10.1001/jamanetworkopen.2024.45995)
Supplement: Supplement 2. — Data Sharing Statement [file jamanetwopen-e2445995-s002.pdf]

Data Sharing Statement

Data
Data available: No

Additional Information
Explanation for why data not available: Both the HRSA Quarterly Summary and the NHSC field strength data are publicly available at https://data.hrsa.gov/data/downloadand
